# Strategies of LncRNA DLX6-AS1 on Study and Therapeutics

**DOI:** 10.3389/fgene.2022.871988

**Published:** 2022-06-01

**Authors:** Yanyan Zhao, Pei Li

**Affiliations:** Department of Pathophysiology, School of Basic Medical Sciences, Zhengzhou University, Zhengzhou, China

**Keywords:** lncRNA DLX6-AS1, regulatory mechanism, tumor, novel research methodologies, therapeutic strategies

## Abstract

Accumulating evidence has revealed the vital regulatory roles of lncRNA DLX6-AS1 in various tumors at pre-transcriptional, transcriptional, and post-transcriptional levels, which makes it a potential prognosis factor and therapeutic target. In addition, the presence of lncRNA DLX6-AS1 in the exosomes of peripheral blood of patients with tumors may also contribute to it being a possible cancer-related biomarker. However, most literature studies are devoted to studying the effect of lncRNA DLX6-AS1 as a sponging molecule of miRNAs, the research of which is likely to get stuck into a dilemma. Literature studies published already have demonstrated an exciting cell malignant phenotype inhibition with the knockdown of lncRNA DLX6-AS1 in various tumor cell lines. With the comprehensive development of delivery systems, high-throughput sequencing, and aptamers, the problems of finding novel research methods and exploring the therapeutic options which are based on lncRNA DLX6-AS1 *in vivo* could come into a period to deal with. This review aims to summarize the research statuses of lncRNA DLX6-AS1, discuss other study methodologies and therapeutic strategies on it, which might be of help to the deep learning of lncRNA DLX6-AS1 and its application from basic to clinical research.

## 1 Introduction

Long non-coding RNAs (lncRNAs) are a class of non-coding RNAs with a length longer than 200 nucleotides, which lack the capability of encoding proteins and were transcriptional noises at first. lncRNAs are transcribed from the opposite strand of the protein-coding genes and overlap one-third of them. lncRNA DLX6-AS1 is usually dysregulated in many tumor tissues and cell lines. Besides, the capability of lncRNA DLX6-AS1 to bind with DNAs, RNAs, and proteins makes its regulatory roles represent at pre-transcriptional, transcriptional, and post-transcriptional levels. Increasing evidence has implied the crucial regulatory roles of lncRNA DLX6-AS1 in various tumors, demonstrating the crucial influence of lncRNA DLX6-AS1 on tumorigenesis and development and making it a candidate biomarker to assist treatment and diagnosis of tumors.

## 2 lncRNA DLX6-AS1 and Its Spliced Form Evf2

lncRNA DLX6-AS1, located at the human chromsome7q21.3 (GRCh38/hg38: chr7: 96955141-97014065) with a length of 1990 base pairs, is the reverse transcript of the upstream of the Dlx6 gene. In mouse, lncRNA DLX6-AS1 is also called Evf1 (embryonic ventral forebrain1) and is located at chromosome 6 (Kohtz and Fishell, 2004). Evf1 is transcribed from the upstream of the DLX6 gene, which comprises two exons with about a 37.5 kb intronic region between them. It was first found as one of the target genes of the sonic hedgehog in the ventral forebrain (Kohtz and Fishell, 2004). Its spliced form Evf2 (also known as DLX6OS1) is transcribed from the ultra-conserved region of the DLX5/6 ei enhancer and comprises three parts which can be divided into the Evf2 5′ unique region, the Evf2 3′ unique region, and the common region with Evf1 (Feng et al., 2006). It has been found that the regulatory roles of Evf2 involve DNA methylation, histone deacetylation, and chromosome topological changes.

### 2.1 The Regulatory Roles of Evf2

#### 2.1.1 DNA Methylation and Histone Deacetylation

**FIGURE 1:**
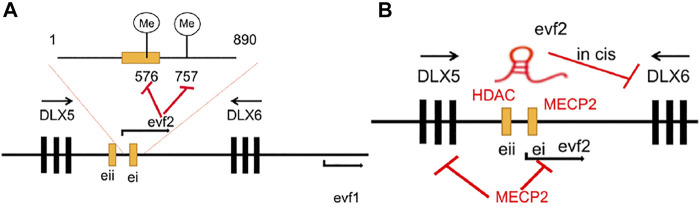
**(A)** Evf2 induces ^576^CpG and ^757^CpG site methylation in the ultra-conserved region of DLX5/6 ei/eii. **(B)** Evf2 recruits MECP2 and further absorbs HDAC1 into the DLX5/6 ei/eii region to repress DLX5 and DLX6 gene expression.

Berghoff et al. (2013) found that Evf2 induced site-specific methylation of Dlx5/6 ei CpG^576^ and CpG^757^ in the E13. 5 MGE of the Evf2 transgene mice (Evf2^TS/TS^; R) but did not affect the expression levels of Dlx5 and Dlx6 when the expression levels of evf2 were at 0.38 x wild-type levels. Apart from regulating gene methylation, it was also revealed by Bond et al. (2009) that Evf2 could recruit MECP2 to the conserved region of DLX5/6 ei/eii which decreased the recruitment of HDAC1 in the DLX5/6eii, and thus, may be the reason for the increased expression level of Dlx5 and Dlx6. Besides, Cajigas et al. (2015) also found that Evf2 lncRNAs could form an Evf2-DLX1 ribonucleoprotein (RNP), which contained the SWI/SNF-related chromatin remodelers Brahma-related gene 1 (BRG1, SMARCA4) and Brahma-associated factor (BAF170, SMARCC2) in the DXL5/6 ei region of a developing mouse forebrain, to regulate chromatin remodeling and gene expression. They revealed that Evf2 increased BRG1 binding with key Dlx5/6 enhancers with changes in lysine acetylation of histones H3 and H4, which led to significantly reduced H3AcK9 and H3AcK18 at ei, reduction of H3AcK18 to a lesser extent at eii, and an H4AcK5 decrease at four sites, while total H4AcK decreased at three intergenic sites of Evf2^TS/TS^ E13.5GE when compared with the decrease at those of EVF2^**+/+**^ E13.5GE (Cajigas et al., 2015) (Figure 1).

#### 2.1.2 Chromosome Topological Changes

Chromosome topological changes allow the regulatory effects of enhancers to display over mega-base distances (Mir et al., 2019). Evf2, a cloud-forming DLX5/6 ultra-conserved enhancer ei lncRNA, can directly interact with its targeting genes and regulate the topology across a 27-Mb region by altering the gene–distance relationships among Dlx5/6, Umad1, and Akr1b8 genes (Cajigas et al., 2018). It was also found that evf2 could regulate the Dlx5/6UCE interactions to regulate the Rbmb8 gene repression *via* inducing the formation of protein pools (Sox2, Dlx, and Smarca4) (Cajigas et al., 2015).

#### 2.1.3 Transcriptional Regulation

Berghoff et al. (2013) found that evf2 can regulate evf2, DLX6, and DLX5 expression *via* the competitive binding of MECP2 and DLX1/2 to the DLX5/6 ei/eii region, and the repression of the DLX6 gene is via its antisense transcription. The antagonism of MECP2 and Dlx1/2 comparatively binding to the ultra-conserved region leads to three kinds of activity statuses of the DLX5/6 ei/eii region, which regulates evf2 and Dlx5 differentially: inactive (MECP2 binds two gene loci), low activity (MECP2 and DLX1/2 bind one gene locus), and high activity (Dlx1/2 occupies two gene loci) (Berghoff et al., 2013). Feng et al. (2006) also demonstrated that evf2 could serve as the coactivator of DLX2 to facilitate the expression of DLX5 and DLX6 genes.

## 3 lncRNA DLX6-AS1 in Tumors

### 3.1 Pre-Transcriptional Regulation

#### 3.1.1 Mediating Methylation

Zhao et al. (2020) found that overexpression of lncRNA DLX6-AS1 could recruit DNA methyltransferase 1 (DNMT1) to the promoter region of its downstream target gene LARGE, which promoted the progression and lymph node metastasis of prostate cancer. It was also discovered by Zhao and Xu (2020) that lncRNA DLX6-AS1 was upregulated in endometrial cancer tissues and cell lines. It could form an RNA–DNA triplex *via* its triplex-forming oligonucleotide (TFO) sequence and the DLX6 triplex target site (TTS). Besides, lncRNA DLX6-AS1 also recruited a transcription factor E2F1 and histone acetyltransferase p300 to the promoter region of DLX6, which enhanced DLX6 expression and promoted proliferation, invasion, and reduced apoptosis of endometrial cancer cell lines (Zhao and Xu, 2020).

Maja Olsson and his colleagues (Olsson et al., 2016) also reported that lncRNA DLX6-AS1, one of the hyper-methylated genes in NB, was over-expressed and indicated a poorer prognosis. However, it is well known that hyper-methylation reduces transcriptional activity suppression, so the underlying molecular mechanisms need further exploration.

#### 3.1.2 Interaction With Transcription Factors (Self-Regulation)

Lin et al. (2021) revealed that the hyper-methylated status of the promoter region of the DLX6-AS1 gene might be a novel progression-related and prognostic marker for colorectal cancer. Intriguingly, the methylation of the gene promoter region usually leads to transcriptional silencing. However, the expression level of lncRNA DLX6-AS1 in colorectal carcinoma cell lines and tissues is upregulated. Hence, there must be some other underlying mechanism that remains unknown. Besides, Zhao et al. (2021) also demonstrated that H3K4me1 can induce histone methylation around the DLX6-AS1 promoter region, which could upregulate the expression level of lncRNA DLX6-AS1 in lung squamous cell carcinoma cell lines and lead to cisplatin resistance.

### 3.2 Post-Transcriptional Regulation

#### 3.2.1 Serving as a ceRNA

LncRNA DLX6-AS1 plays key regulatory roles in regulating tumorigenesis and progress via many mechanisms. One of the most important mechanisms is formation of lncRNA DLX6-AS1-miRNAs-mRNAs ceRNAs regulatory networks, in which lncRNA DLX6-AS1 serves as an endogenous competing RNA to competitively sponge miRNAs to further up-regulate the downstream target genes (mRNAs) of miRNAs (Zeng et al., 2017; Zhang et al., 2017; An et al., 2018; Zhangmou et al., 2018; Li X. et al., 2019; Yang Q. et al., 2019; Zhang H. Y. et al., 2019; Li D. et al., 2019; Yang J. et al., 2019; Zhang N. et al., 2019; Fang et al., 2019; Huang et al., 2019; Lei et al., 2019; Sun et al., 2019; Wang et al., 2019; Zhao et al., 2019; Liu Y. et al., 2020; Du et al., 2020; Hu et al., 2020; Jia et al., 2020; Kong et al., 2020; Kong and Zhang, 2020; Liang et al., 2020; Qian et al., 2020; Wang et al., 2020; Xie et al., 2020; Yang et al., 2020; Liu et al., 2021; Wang et al., 2021; Wu et al., 2021; Zhao et al., 2021; Zheng et al., 2021; Zhu et al., 2021). Xue et al. (2021) and Feng et al. (2021) also reviewed the regulatory roles serving as a ceRNA in various tumors which influence the prognosis of patients and which may be the therapeutic and prognosis target in malignant tumors. Table 1 summarizes the microRNAs sponged by lncRNA DLX6-AS1 in various tumors.

**TABLE 1:** miRNAs sponged by lncRNA DLX6-AS1 and miRNA targets in various tumors.

miRNAs	Tumors	mRNAs	References
miR-16-5p	CC	ARPP19	Xie et al. (2020)
miR-199a	CC	—	Wang et al. (2019)
miR-26a	CRC RCC LC	EZH2 PTEN TRPC3	Zeng et al. (2017); Liu Y. et al. (2020); Kong et al. (2020)
miR-27b-3p	NSCLC	GSPT1	Sun et al. (2019)
miR-181a-5p/miR-382-5p	LUSC	CELF1	Zhao et al. (2021)
miR-107	NB	BDNF	Zhang H. Y. et al. (2019)
miR-506-3p	NB	STAT2	Hu et al. (2020)
miR-513c-5p	NB	PLK4	Jia et al. (2020)
miR-124-3p	ES(2)	CDK4	Lei et al. (2019)
miR-129-5p	OS	DLK1	Zhangmou et al. (2018)
miR-144	NSCLC	PRR11	Huang et al. (2019)
miR-16-5p	NSCLC	BMI1	Wu et al. (2021)
miR-181b	PC	ZEB2	An et al. (2018)
miR-497-5p	PC	FZD4/FZD6/Wnt/beta-catenin	Yang J. et al. (2019)
miR-195-5p	OC	FLH2	Kong and Zhang, (2020)
miR-197-5p	Glioma	E2F1	Li X. et al. (2019)
miR-199a-5p	NPC	HIF-1α	Yang et al. (2020)
miR-199b-5p	TNPC	Paxillin	Du et al. (2020)
miR-203a	HCC	MMP-2	Zhang et al. (2017)
miR-15a-5p	HCC	CXCL17	Wang et al. (2021)
miR-513c	HCC	Cul4A/ANXA10	Liu et al. (2021)
miR-424-5p	HCC	WEEK1	Li D. et al. (2019)
miR-204-5p	GCC	OCT-1	Liang et al. (2020)
miR-4290	GCC	PDK1	Qian et al. (2020)
miR-223	BC(1)	HSP90B1	Fang et al. (2019)
miR-195-5p	BC(1)	VEGFA	Wang et al. (2020)
miR-376c	LC	—	Yang Q. et al. (2019)
miR-505-3p	BC(2)	RUNX2	Zhao et al. (2019)
miR-641	OS	HOXA9	Zhang N. et al. (2019)
miR-497-5p	PC(2)	SNCG	Zhu et al. (2021)
miR-193b-3p	TC	HOXA1	Zheng et al. (2021)

NSCLC, non-small cell lung cancer; LUSC, lung squamous cancer; GCC, gastric cancer; CRC, colorectal cancer; ESCC, esophageal squamous carcinoma; HCC, hepatocellular carcinoma; PC, pancreatic cancer; EC(1),esophageal carcinoma; RCC, renal cell carcinoma; BC(1), bladder cancer; CC, cervical cancer; OC, ovarian cancer; LC, laryngeal carcinoma; NPC, nasopharyngeal carcinoma; OS, osteosarcoma; ES(2), Ewing’s sarcoma; NB, neuroblastoma; BC(2), breast cancer; PC(2), prostate cancer; TC, thyroid cancer.

#### 3.2.2 Stabilizing mRNAs

The incidence and mortality of gastric cancer (GC) rank third and fifth, respectively (Fu et al., 2019), and is usually diagnosed at an advanced stage attributing to a lack of typical symptoms. Hence, it is imperative to find molecular biomarkers to assist early prognosis. Wu et al. (2020) found that lncRNA DLX6-AS1 could be a molecular link to stabilize mRNA levels in tumors. In gastric cancer, lncRNA DLX6-AS1 stabilizes MAP4K1 mRNA levels *via* regulating FUS protein expression, forming FUS-MAP4K1 protein-mRNA complexes, by which lncRNA DLX6-AS1 promotes the cell proliferation, migration, and EMT of gastric cancer.

**FIGURE 2:**
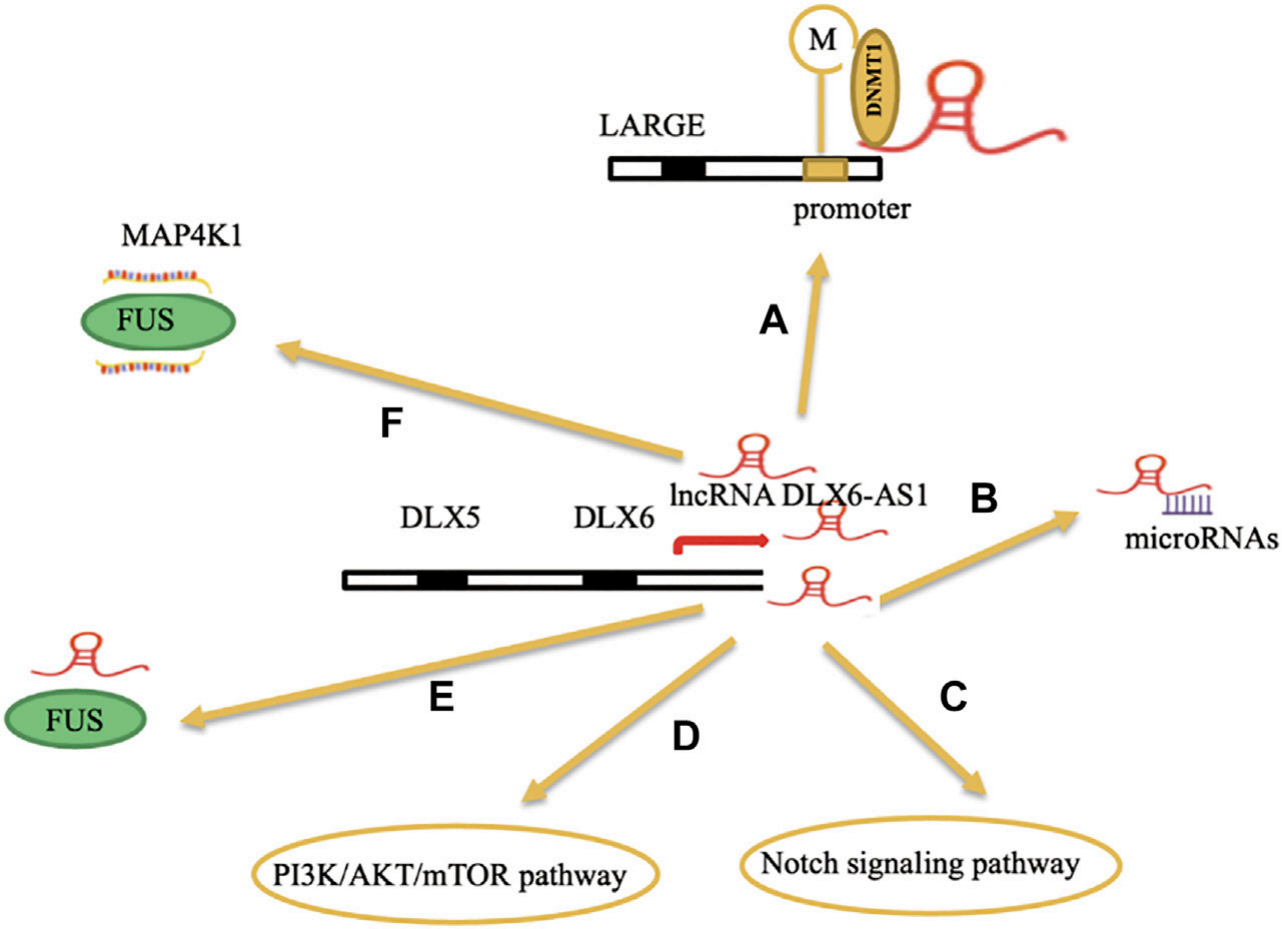
**(A)** Overexpression of lncRNA DLX6-AS1 could recruit DNA methyltransferase 1 (DNMT1) to the promoter region of its downstream target gene LARGE. **(B)** lncRNA DLX6-AS1 acts as an endogenously competing RNA to sponge miRNAs. **(C,D)** lncRNA DLX6-AS1 can regulate the notch signaling pathway and PI3K/AKT/mTOR pathway in epithelial ovarian cancer and colorectal cancer, respectively. **(E)** lncRNA DLX6-AS1 indirectly binds with MAP4K1(mRNA) *via* FUS(protein) to increase the stability of MAP4K1 in gastric cancer. **(F)** lncRNA DLX6-AS1 interacts with FUS (proteins) and regulate its expression levels in breast cancer.

Tian et al. (2020) made a meta-analysis between the expression levels of lncRNA DLX6-AS1 and the clinicopathology and prognosis of various cancers. They found that high expression levels of lncRNA DLX6-AS1 were associated with poor overall survival in tumor patients and overexpression of lncRNA DLX6-AS1 was associated with tumor stage (*p* < 0.01), tumor size (*p* < 0.01), lymph node metastasis (*p* < 0.01), and distant metastasis (*p* < 0.01). Hence, all the aforementioned factors have shown the vital role of research on lncRNA DLX6-AS1 in various tumors (Figure 2).

#### 3.2.3 Crosstalk Between Tumor-Associated Macrophages

Wang et al. (2021) found that lncRNA DLX6-AS1 was upregulated in serum exosome derived from patients with hepatocellular carcinoma (HCC) and the density of TAMs in cancer tissues was higher than that in adjacent tissues. They also demonstrated that HCC-exosomes could be delivered to TAMs in a tumor microenvironment by which DLX6-AS1-overexpressed TAMs could further promote the progression of HCC. In the mechanism study, they revealed that after co-culturing HCC-exosomes with monocyte THP-1 cell lines, THP-1 cells are more polarized into M2 macrophages. In addition, after co-injecting hepatocellular cell lines with primed THP-1 cells (THP-1/HCC-exosomes transferred with oe-DLX6-AS1) into the right liver lobe of the mice, they also discovered that primed THP-1 cells (THP-1/HCC-exosomes transferred with oe-DLX6-AS1) could promote HCC lung metastasis *in vivo*. All the above-mentioned observations show that the crosstalk between lncRNA DLX6-AS1 and tumor-associated macrophages (TAMs) could enhance the progression of HCC, which also may provide novel research clues on the regulatory roles of lncRNA DLX6-AS1 in a tumor microenvironment (TME).

#### 3.2.4 Exosomal lncRNA DLX6-AS1

Exosomes (40–100 nm) are key mediators of cell-to-cell communication by transmitting biomolecules such as mRNAs, miRNAs, and lncRNAs (Skog et al., 2008). Several research studies demonstrated that the expression level of exosome-lncRNA DLX6-AS1 in patients with cancer can be a promising prognosis biomarker in tumors.

Zhang et al. reported that the circulating exosome lncRNA DLX6-AS1 expression levels in the serum of patients with NSCLC were significantly higher than those in healthy donors, and its specificity and sensitivity were higher than that of CYFRA21-1 (Ding et al., 2021), which served as a diagnostic marker in NSCLC. They also found that the expression level of exosome lncRNA DLX6-AS1 was positively correlated with tumor differentiation, TNM stage, and lymph vascular invasion, making it a potential early diagnostic and metastatic marker for NSCLC. Ding et al. enrolled 114 patients with cervical cancer (CC), 60 patients with cervical intraepithelial neoplasia (CIN), and 110 healthy women to their study (Ding et al., 2021). They found that the exosome lncRNA DLX6-AS1 level was elevated in CC patients compared with patients with CIN and normal healthy donors. A high serum exosome lncRNA DLX6-AS1 expression level was positively associated with lymph node metastasis, histopathological differentiation, FIGO stage, and shortened survival of patients with CC (Ding et al., 2021). However, because of the limited number of enrolled patients and high false positive and negative results due to the use of serum exosome lncRNA DLX6-AS1 alone as the prognosis factor of patients with CC, they suggested that a combination with other known tumor biomarkers and clinicopathological parameters was needed to accurately predict the clinical outcome of CC.

### 3.3 Potential Molecular Biomarker in Tumors: lncRNA DLX6-AS1

As a tumor-promoting gene, lncRNA DLX6-AS1 is often upregulated in many tumors compared with normal tissues. Overexpression of lncRNA DLX6-AS1 could mediate the genesis and progression of many tumors, the knockdown of which could alleviate this phenomenon. Besides, high expression levels of lncRNA DLX6-AS1 are also positively associated with the clinicopathological parameters and negatively with the prognosis of patients with a tumor, which could help with the pathological diagnosis, the survival prediction, and histopathological molecular subtyping. Besides mediating the malignant phenotype of tumor cells, over-expressed lncRNA DLX6-AS1 could also be able to communicate with the tumor microenvironment (TME) such as tumor-associated macrophages (TAMs), which could make it highly expressed in cells in the TME via exosomes and further promote the progress of tumors. In addition, the exosome-lncRNA DLX6-AS1 level could be differently derived from the peripheral blood of patients with a tumor diagnosed at different stages. These features might make it a potential prognosis marker combined with other known tumor molecular markers.

## 4 lncRNA DLX6-AS1 in Other Diseases

### 4.1 Post-Transcriptional Regulation

lncRNA DLX6-AS1, also named evf1 in the mouse, which involves the development of the mouse’s embryonic ventral forebrain, is dysregulated in developing human telencephalon modeling autism spectrum disorder (ASD). Mariani et al. (2015) revealed that lncRNA DLX6-AS1 was one of the top ten up-regulated genes in cerebral organoids derived from members of a family with idiopathic ASD at TD11 and TD31. Wang et al. (2017) also found that DLX6-AS1 and DLX1 were two of three DEGs in CHD8^+/-^ (CHD8 is a highly mutated gene in autism spectrum disorders) cerebral organoids, which increased ∼39- and 13-fold, respectively, and were hardly expressed in controls (Wang et al., 2017). In addition, it is well known that evf2 (the spliced form of evf1) forms a complex with DLX1 and DLX2 proteins to facilitate the enhancer activity of DLX5 and DLX6 gene expression. Hence, it supports the potential that lncRNA DLX6-AS1 cooperates with the Dlx family to regulate brain development in humans whose dysregulation is related with nervous system disorders.

lncRNA DLX6-AS1 also serves as a competitively endogenously binding RNA in preeclampsia (PE) and sepsis-induced acute kidney injury (AKI). For instance, Tan et al. (2018) found that high expression levels of lncRNA DLX6-AS1 inhibited proliferation, migration, and invasion of trophoblast JEG3 and HTR-8/SVneo cells *via* directly regulating the miR-376c/GADD45A axis. Interestingly, it has been proven by Liu R. et al. (2020) that the increased lncRNA DLX6-AS1 expression inhibited proliferation, invasion of trophoblast cells of the placenta, and the angiogenesis of HUVEC cells *via* downregulating ERP44 by sponging miR-149-5p in preeclampsia. Tan et al. (2020) reported that the knockout of lncRNA DLX6-AS1 regulated the miR-223-3p/NLRP3 axis in HK-2 cells to induce sepsis-induced acute kidney injury. In addition, Zheng et al. (2022) first demonstrated that over-expressed cAMP-response element binding protein (CREB) induces podocyte injury in diabetic nephropathy via upregulating the lncRNA DLX6-AS1 expression level *in vitro* and *in vivo*.

## 5 Other Study Methodologies of LncRNA DLX6-AS1

Most research studies on lncRNA DLX6-AS1 are related to its sponging function to microRNAs as a competitive endogenous RNA via base pairing to each other. MicroRNAs are single-stranded RNAs and generated from endogenous hairpin-shaped transcripts with a length of 19–25 nucleotides (Kim, 2005); they are predicted to regulate the activity of approximately 50% of all protein-coding genes in mammals (Krol et al., 2010). Because of its significant roles in many tumors as a suppressor gene or tumorigenic gene, therapeutics based on microRNAs or anti-microRNAs have been processed to advanced clinical trial stages. Therefore, this may be one of the important reasons for studying the regulatory function of lncRNA DLX6-AS1 in microRNAs.

Due to the biochemical approaches and high-throughput sequencing (Qian et al., 2019), the secondary structure conformations of lncRNAs have been found, and the relationship of lncRNAs between structures and functions should be considered. Hence, the studies of interactions between lncRNA DLX6-AS1 and RNAs, DNAs, or proteins may provide novel research insights into human tumors. We recommend readers to get detailed research clues and inspiration from the articles written by Qian et al. (2019) and Statello et al. (2021).

## 6 Potential Therapeutic Strategies of lncRNA DLX6-AS1

RNA interference (RNAi) is an endogenous cellular mechanism for regulating gene expression via incorporating siRNAs into RNA-induced silencing complexes to mediate target genes’ cleavage and degradation (Tabernero et al., 2013). lncRNA DLX6-AS1 is usually upregulated in many solid tumor tissues compared to adjacent normal tissues playing a tumorigenic gene role. It is reported that lncRNA DLX6-AS1 is upregulated in many tumor cell lines, and its knockdown can significantly suppress the malignant phenotype of tumor cell lines.

Novel delivery systems can successfully deliver miRNAs to tumor locations without being degraded by RNase in blood and show obvious adverse effects in patients, encapsulating si-lncRNA DLX6-AS1 in lipid nanoparticles to deliver them to tumor sites using the EPR (enhanced permeability and retention) effect on tumor locations (Matsumura et al., 1986). We can also encapsulate si-lncRNA DLX6-AS1 in specific aptamers or target motif-modified nanoparticles to deliver it to tumor locations dependent on ligand–receptor interaction. Simply inhibiting tumor cell malignant phenotypes by delivering si-lncRNA DLX6-AS1 to tumor sites may not show significant tumor inhibitory effects on patients at the advanced stages. Hence, we can encapsulate multiple siRNAs and cooperate with precise photodynamic or photo-thermal therapies to get significant therapeutic effects. However, due to the discontinuous vascular epithelium of the kidneys and spleen similar to immature vascular epithelium in tumor sites, nanoparticles also accumulate easily in these organs and thus may contribute to adverse effects. Nanoparticle drug formulations and pre-clinical trials should be rigorously designed.

## 7 Summary

Increasing evidence has implied the crucial regulatory roles of lncRNA DLX6-AS1 in various tumors which mainly represent on pre-transcriptional and post-transcriptional levels. One of the most prevalent roles is its sponging function as a ceRNA to form regulatory networks to influence cancer cells’ biological characteristics. Besides, LncRNA DLX6-AS1 can also facilitate mRNA stability to influence cell behaviors in tumors and bind with the DNA promoter region to promote methylation of DNAs which further inhibits DNA transcription. Interestingly, in tumors, the upregulation of lncRNA DLX6-AS1 mostly promotes cell proliferation, invasion, and migration, which is the opposite in preeclampsia. Wang et al. (2017) also found that DLX6-AS1 and DLX1 are dysregulated in CHD8^+/-^ cerebral organoids, supporting its developmental regulatory role in human brain development and nervous disorders. The novel biochemical approaches and high-throughput sequencing can provide new insights into the study of the interactions between lncRNA DLX6-AS1 and RNAs, DNAs, or proteins. Along with the rapid development of delivery systems and precisely cooperated therapies, it may provide the possibility of pre-clinical therapeutics based on lncRNA DLX6-AS1 in many tumors.
